# Supplementary material for: plsMD: a plasmid reconstruction tool from short-read assemblies
Source: BMC Bioinformatics. 2026 Aug 13;27:181. doi: 10.1186/s12859-026-06585-y (PMC13474732; doi:10.1186/s12859-026-06585-y)

Supplementary Figure S1. Representative structural comparisons between long-read-derived ground-truth plasmids and plsMD reconstructions across multiple replicon types. Comparison of fully closed ground-truth plasmids and their corresponding plsMD reconstructions from the same isolates, representing four replicon types: (a) IncFIB(pNDM-Mar), (b) IncL/M (pOXA-48), (c) IncA(C2), and (d) IncFIB(K)_Kpn3. Plasmids are visualized using Proksee and annotated for plasmid replicons, antimicrobial resistance (AMR) genes, virulence factor (VF) genes, and insertion sequences (ISs). The comparisons illustrate preservation of the overall plasmid backbone, gene organization, and the distribution of key genetic features between experimentally resolved ground-truth plasmids and the corresponding plsMD reconstructions.

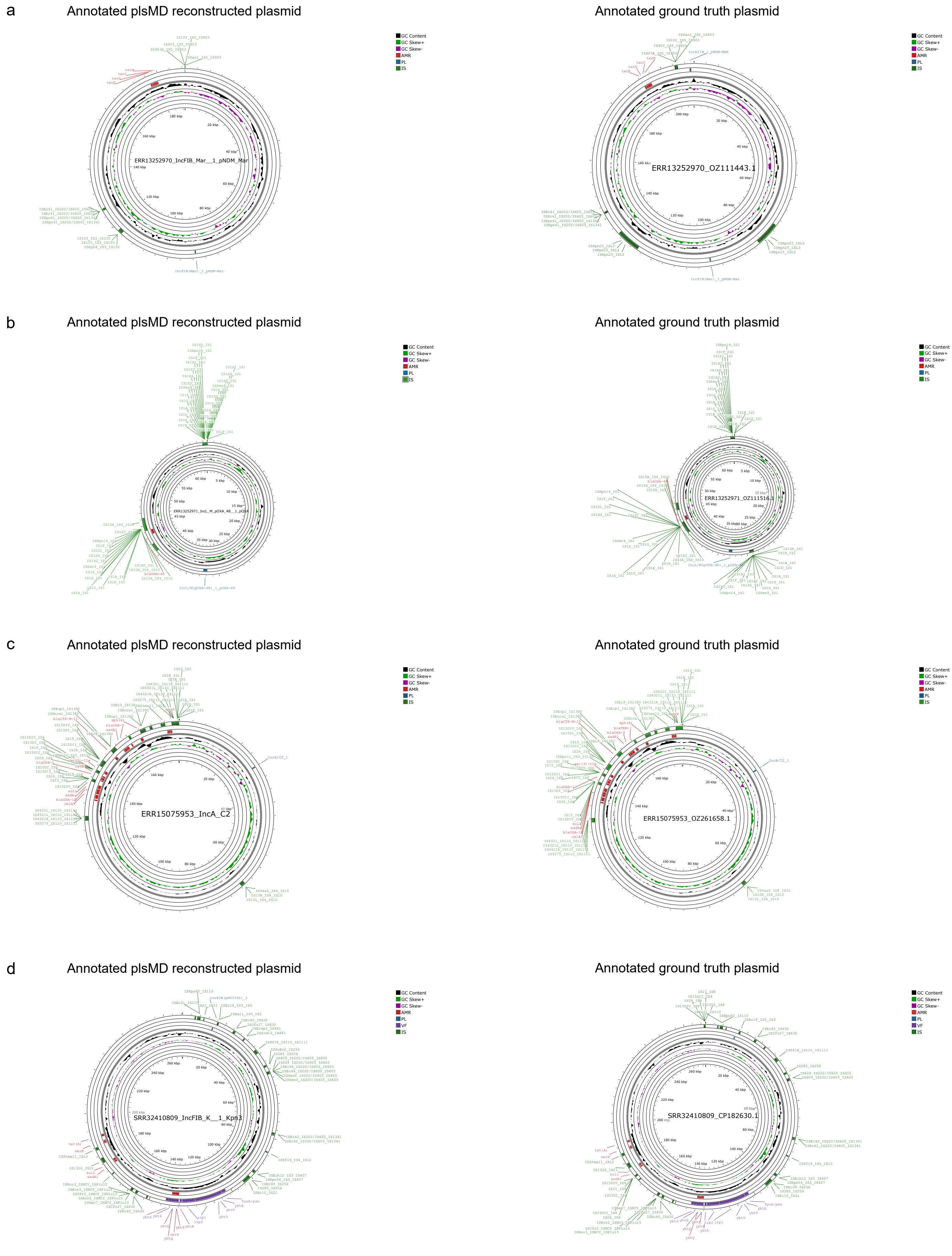

Supplement: Supplementary file 1 [file 12859_2026_6585_MOESM1_ESM.pdf]
